# Early Feasibility Study of a Hybrid Tissue-Engineered Mitral Valve in an Ovine Model

**DOI:** 10.3390/jcdd11020069

**Published:** 2024-02-19

**Authors:** Ramin Zareian, Samuel D. Zuke, Daisuke Morisawa, Roger S. Geertsema, Mariwan Majid, Clinton Wynne, Jeffrey C. Milliken, Arash Kheradvar

**Affiliations:** 1 Department of Biomedical Engineering, University of California, Irvine, CA 92697, USA; 2 University Laboratory Animal Resources, Office of Research, University of California, Irvine, CA 92697, USA; 3 Division of Cardiothoracic Surgery, UC Irvine Medical Center, Orange, CA 92868, USA; 4 GE Healthcare, Waukesha, WI 53188, USA

**Keywords:** hybrid tissue-engineered heart valve, nondegradable scaffold, surgical heart valve implantation

## Abstract

Tissue engineering aims to overcome the current limitations of heart valves by providing a viable alternative using living tissue. Nevertheless, the valves constructed from either decellularized xenogeneic or purely biologic scaffolds are unable to withstand the hemodynamic loads, particularly in the left ventricle. To address this, we have been developing a hybrid tissue-engineered heart valve (H-TEHV) concept consisting of a nondegradable elastomeric scaffold enclosed in a valve-like living tissue constructed from autologous cells. We developed a 21 mm mitral valve scaffold for implantation in an ovine model. Smooth muscle cells/fibroblasts and endothelial cells were extracted, isolated, and expanded from the animal’s jugular vein. Next, the scaffold underwent a sequential coating with the sorted cells mixed with collagen type I. The resulting H-TEHV was then implanted into the mitral position of the same sheep through open-heart surgery. Echocardiography scans following the procedure revealed an acceptable valve performance, with no signs of regurgitation. The valve orifice area, measured by planimetry, was 2.9 cm^2^, the ejection fraction reached 67%, and the mean transmitral pressure gradient was measured at 8.39 mmHg. The animal successfully recovered from anesthesia and was transferred to the vivarium. Upon autopsy, the examination confirmed the integrity of the H-TEHV, with no evidence of tissue dehiscence. The preliminary results from the animal implantation suggest the feasibility of the H-TEHV.

## 1. Introduction

At present, there are primarily two types of artificial heart valves—mechanical and bioprosthetic—used to replace a diseased native valve. Mechanical heart valves (MHVs) are the recommended choice for younger patients, primarily due to their durability [1]. Yet, a significant drawback of these valves is the lifelong requirement for anticoagulant medication. On the contrary, bioprosthetic heart valves (BHVs), crafted from either porcine or bovine tissue, may not necessitate anticoagulant medications owing to their biocompatible surface and enhanced blood flow dynamics. Nonetheless, recent studies indicate the potential need for anticoagulant medications even in patients with BHVs [2]. In contrast to MHVs, BHVs exhibit lower risks of thrombogenicity and superior hemodynamics, offering significant advantages for older patients. However, BHVs are prone to calcification and exhibit a limited durability due to their progressive degeneration, with the rate of degeneration inversely correlated with the patient’s age at the time of implantation [3,4]. As a result, younger patients requiring a heart valve replacement are confronted with the challenging decision between two equally undesirable therapeutic options: MHVs requiring lifelong anticoagulant medications or BHVs with a limited durability and the necessity for an eventual replacement. Existing guidelines advise the use of BHVs exclusively for patients aged 60 and above, citing a greater likelihood of degeneration and calcification in younger patients [5].

During the past decade, tissue-engineered heart valves (TEHVs) have emerged as a promising solution to replace diseased heart valves for both young and elderly patients. Nevertheless, the current TEHVs face limitations in withstanding the hemodynamic loads, particularly in the left ventricle [6,7]. A noteworthy hurdle associated with TEHVs involves the contraction and migration of host smooth muscle cells (SMCs), resulting in a reduction in the size of the tissue constructs around the valve leaflets [8,9,10]. Utilizing degradable scaffolds, including synthetic polymers [7,11,12,13] and entirely biological materials [10,14], in current TEHVs results in poor leaflet coaptation, progressive regurgitation, and valvular insufficiency.

**Figure 1:**
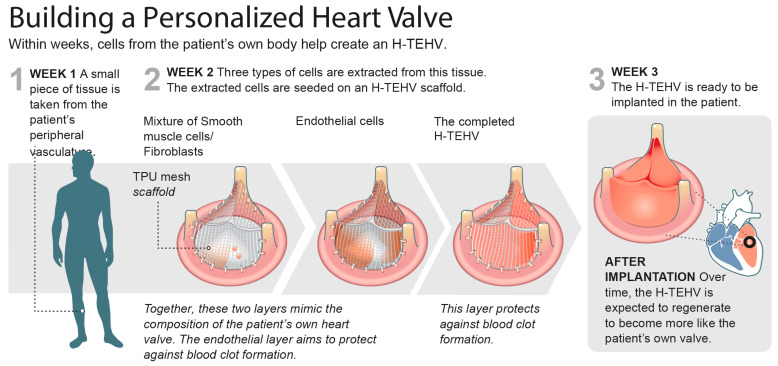
Illustrated above are the three key steps involved in developing an H-TEHV. In this initial step, a tissue segment is extracted from the subject’s vessel (jugular vein for sheep and ultimately saphenous vein for humans). The harvested tissue is then processed to extract and categorize the subject’s own cells into three distinct lines: smooth muscle cells (SMCs), fibroblasts (FBs), and endothelial cells. The H-TEHV scaffold undergoes coating with two different cell layers. The first layer consists of a mixture of SMCs and FBs suspended in a collagen gel, while the second layer involves incorporating endothelial cells into a collagen gel. Once both layers are applied, the completed H-TEHV is placed in an incubator for further development. After a three-week incubation period, the autologous H-TEHV is prepared for implantation. This final step involves implanting the developed H-TEHV back into the same subject from whom the tissue was initially harvested.

In our pursuit to ultimately address these challenges, we have been developing hybrid tissue-engineered heart valves (H-TEHVs). Unlike BHVs, H-TEHVs are primarily comprised of live autologous tissue harvested from the same subject, eliminating the need for blood-thinning medication as required with MHVs. The concept of an H-TEHV is grounded in utilizing a nondegradable mesh as the valve scaffold and then enclosing it with various autologous cell types—vascular smooth muscle cells (SMCs), vascular fibroblast (FB) cells, and endothelial cells (ECs)—to serve the functions of valvular interstitial cells (VICs) and valvular endothelial cells, respectively (see Figure 1) [15,16,17]. While this approach may not accommodate growth along with the subject, making it less suitable for the pediatric age group with developing hearts, its permanent scaffold addresses the scaffold degradation issues commonly observed in TEHVs. The present work describes an early feasibility study of implanting an H-TEHV in an ovine model.

## 2. Material and Methods

**Figure 2:**
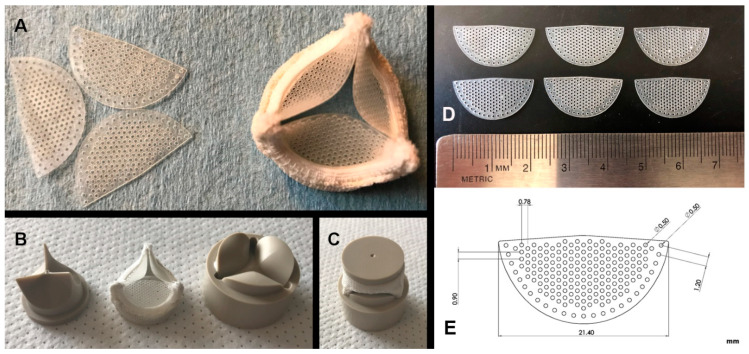
(**A**) On the left side, Carbothane mesh leaflets precisely cut by laser are shown, while on the right side, an assembled heart valve scaffold composed of Carbothane mesh leaflets and a titanium frame are shown; (**B**) the upper and lower segments of the PEEK mandrels matched for a specific size of a Carbothane mesh scaffold; (**C**) the heart valve with a Carbothane mesh scaffold positioned within the assembled PEEK mandrels; (**D**) Carbothane leaflet mesh laser cut according to the computer-aided design (**E**). Dimensions are specified in millimeters.

**H-TEHV scaffold:** We used a leaflet scaffold crafted from Carbothane (AC-4095A), a medical-grade, aromatic polycarbonate-based thermoplastic polyurethane (TPU). The mechanical properties of Carbothane AC-4095A offer a crucial balance of flexibility and durability, which is essential for the functionality of a heart valve. Carbothane has demonstrated a relatively low tendency to calcification, making it a desirable material for cardiovascular implant applications [18]. The Carbothane mesh leaflets were meticulously designed using SolidWorks (Dassault Systèmes, Vélizy-Villacoublay, France) and precisely cut from 250-micron-thick strips of Carbothane by laser to achieve the desired shape, creating a valve scaffold with a 21 mm orifice diameter, as illustrated in Figure 2. These Carbothane leaflets were carefully sewn onto a 3D-printed titanium frame to construct a trileaflet valve scaffold, as shown in Figure 2. The titanium valve frame was covered with surgical fabric, acting as a protective layer and facilitating surgical implantation at the mitral annulus.

**Figure 3:**
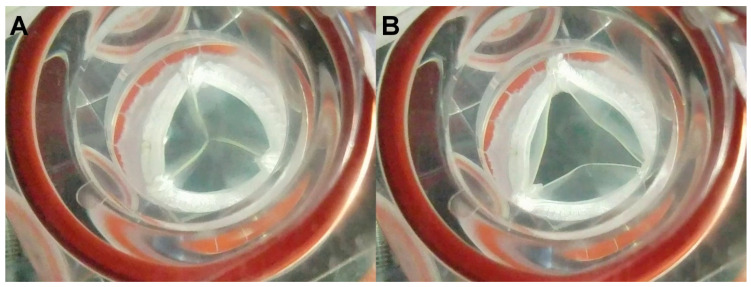
The valve scaffold, constructed from nonmesh Carbothane valve leaflets, is shown in both the fully closed position (**A**) and the open position (**B**) within the AWT system.

**Accelerated wear testing:** We assessed the durability of the 21 mm Carbothane valve scaffolds (*n* = 2) using an accelerated wear testing (AWT) system (M6 heart valve durability tester, Dynatek Labs, Galena, MO, USA). The nonmesh polymeric valve scaffolds were securely mounted in the appropriate test fixtures and introduced into the M6 heart valve durability tester, as illustrated in Figure 3. The valves were submerged into a normal saline solution and maintained at 37 °C throughout the testing period. Achieving full opening and closure, the valves were subjected to 800 cycles per minute (CPM). The images captured from the AWT system depict the full closure (Figure 3A) and opening (Figure 3B) of the valve scaffold. The peak differential pressure during valve closure was consistently maintained at 120 mmHg. The testing spanned 50 million cycles took nearly 45 days, approximating 15 months of exposure in a human heart. Subsequently, both valves were carefully examined under microscope to evaluate the conditions of the leaflets, sutures, and posts after completing the 50 million cycles.

**Figure 4:**
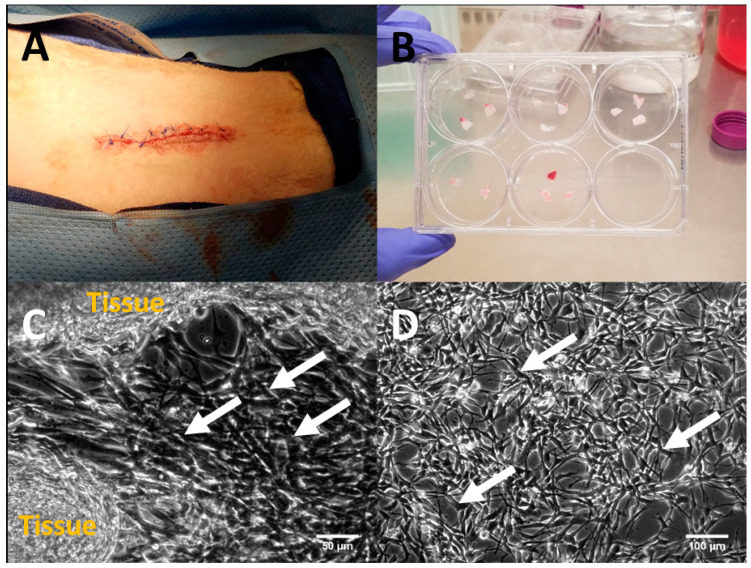
(**A**) Illustration of the jugular vein extraction site on the animal’s neck; (**B**) minced jugular vein tissue were cultured on a sterile Petri dish in the cell culture hood; (**C**) after 5 days in an incubator, cells migrated from the tissue, indicated by white arrows; (**D**) migrated and proliferated cells in a sterile Petri dish, highlighted by white arrows. Scale bars are 50 µm in (**C**) and 100 µm in (**D**), respectively.

**Tissue harvesting and cell extraction/expansion:** Sheep were chosen as the animal model for H-TEHV studies, given their current status as the preferred model for investigating heart valve durability [19]. An approximately 2.5 cm long segment of the jugular vein was surgically excised from a juvenile castrated male sheep (Figure 4A) under anesthesia in a sterile environment at the UCI Medical Center, according to the IACUC protocol #2012-3071. After a complete recovery, the sheep was transferred to the animal care facility for up to three weeks to allow for the preparation of the cultured H-TEHV for implantation. The harvested tissue was promptly transported to our cell culture lab for further processing.

Upon arrival, the jugular vein was finely minced under a sterile cell culture hood and gently washed with sterilized PBS 1X to eliminate blood clots and tissue clumps. The tissue clusters were then cut into 5 mm × 5 mm segments and transferred to a sterilized Petri dish (Figure 4B). The dish was left in the hood for 30 min to allow the tissue fragments adhering to the top of the Petri dish. After this period, a preheated, enriched cell culture medium containing growth factors for endothelial cells, smooth muscle cells, and fibroblasts (4 mL medium for each well) was gently added to each well of the Petri dish (Figure 4B).

The enriched cell culture medium was a combination of three different media:

(1) Endothelial cell growth medium (EGM-2, Lonza CC-3162) enriched with FBS (10.00 mL), hydrocortisone (0.20 mL), hFGF-B (2.00 mL), VEGF (0.50 mL), R3-IGF-1 (0.50 mL), ascorbic acid (0.50 mL), hEGF (0.50 mL), GA-1000 (0.50 mL), and heparin (0.50 mL);
(2) Fibroblast growth medium (FGM-2, Lonza CC-3132) enriched with insulin (0.50 mL), hFGF-B (0.50 mL), GA-1000 (0.50 mL), and FBS (10.00 mL);
(3) Smooth muscle cell growth medium (SmGM-2, Lonza CC-3182) enriched with insulin (0.50 mL), hFGF-B (1.00 mL), GA-1000 (0.50 mL), FBS (25.00 mL), and hEGF (0.50 mL).

The Petri dish was maintained in a 37 °C incubator for one week, with the medium being replaced with preheated, fresh medium every day. After 5 days, the cells migrated out of the tissue fragments, as confirmed under microscope (Figure 4C). Subsequently, we allowed the cells to proliferate in the incubator until they reached a count of 5 million cells for the H-TEHV tissue culture (Figure 4D). Finally, the cells were trypsinized for sorting and characterization as outlined below.

**Cell sorting and characterization:** The trypsinized cells were quantitatively sorted following the procedure outlined by Weber et al. [20], employing a magnetic labeling technique that facilitates sorting through magnetic-activated cell sorting (MACS). Utilizing both positive and negative selections, the method has been used for cell isolation, involving two sequential steps. Initially, vascular endothelial cells were isolated by incubating the extracted cells with CD31 antibody (0.1 mg/mL CD31 antibody-IgG2a, Bio-Rad Laboratories Inc., Hercules, CA) targeting endothelial cells at 20 °C for 30 min. The cells treated with CD31 antibody were then incubated with coated magnetic microbeads (anti-mouse IgG, No. 130-048-402; Miltenyi Biotec, Bergisch Gladbach, Germany) at 4 °C for 15 min. Subsequently, these conjugated endothelial cells were isolated and removed using a magnetic column (positive selection).

The remaining cells, constituting a mixture of SMCs and FBs, were collected in separate cell culture Petri dishes. Throughout all cell culture procedures, the endothelial cells were nourished with an endothelial cell growth medium (EGM-2 Bulletkit CC-3162, Lonza, Allendale, NJ, USA), while the mixture of SMCs/FBs received a combination of 50% smooth muscle growth medium (SmGM-2 Bullekit CC-3182) and 50% fibroblast growth medium (FGM-2 Bullekit CC-3132, Lonza Group, Basel, Switzerland).

**Figure 5:**
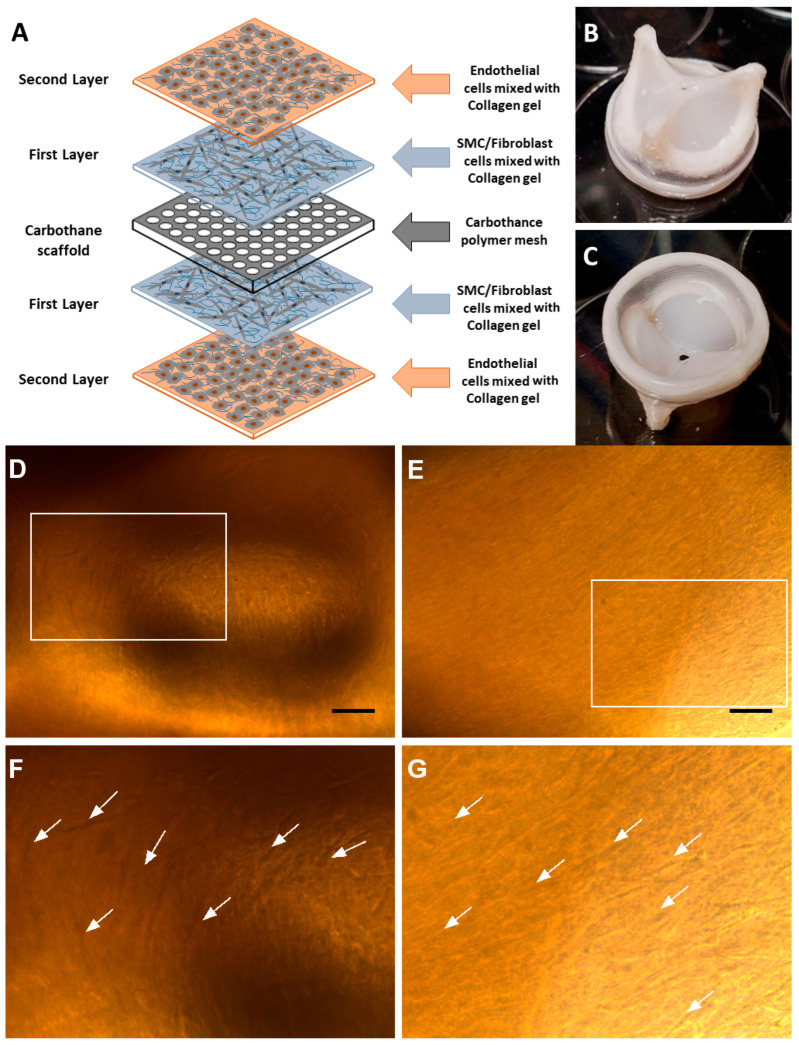
(**A**) A schematic representation of the H-TEHV concept, illustrates the layers of cells mixed with collagen type I on both the top and bottom surfaces of the Carbothane mesh. The orange and blue layers represent endothelial cells and SMCs/FBs mixed with collagen type I gel, respectively, with the central gray plate representing the Carbothane mesh. (**B**) Top and (**C**) bottom views of the fully developed H-TEHV before surgical implantation. (**D**,**E**) present macroscopic views highlighting elongated FBs and SMCs in the initial layer of tissue grown over the valve scaffold. The scale bar for both images is 100 µm. (**F**,**G**) depict magnified areas (originating from a white box in the images (**D**,**E**), respectively), with white arrows indicating the presence of SMCs/FBs in the collagen gel.

**H-TEHV tissue culture:** The Carbothane valve scaffold was coated with a mixture of the sorted cells and type I collagen. Figure 5A provides a schematic representation of the tissue layers on both sides of the Carbothane valve scaffold. This coating process was facilitated using a mandrel apparatus made of biocompatible PEEK (polyether ether ketone) as shown in Figure 2B,C [21]. The trileaflet H-TEHV was created by initially applying a layer of collagen solution mixed with SMCs and FBs over the Carbothane mesh scaffold. This was followed by covering the construct with endothelial cells mixed with collagen. The mandrel apparatus played a crucial role in shaping and stabilizing the cells and tissue layers as they developed in three dimensions to form the heart valve. Figure 2B,C illustrates the mandrel apparatus, which consists of two distinct components that secure the combination of the scaffold and the enveloping tissue layers [21].

Cells combined with collagen were injected onto the scaffold through the hole in the upper component of the mandrel apparatus. This apparatus enables the space adjustment between its two components, accommodating different cell types and tissue layers in sequential steps. Initially, the Carbothane scaffold underwent sterilization with 70% ethanol and was coated with a 1 µg/mL concentration of fibronectin solution. A mixture of SMCs and FBs (5 million cells) was uniformly mixed with collagen type I (RatCol rat tail type I collagen 4 mg/mL, Advanced BioMatrix, Carlsbad, CA, USA) and then injected over the scaffold through the mandrel apparatus (as shown in Figure 2C).

The scaffold within the mandrel apparatus was placed in an incubator at 37 °C for 1.5 h without cell culture media to complete the coating of the H-TEHV’s scaffold’s top and bottom by the SMC/FB mixture. Subsequently, the H-TEHV was transferred into a sterile Petri dish with a mixture of 50% SmGM-2 and 50% FGM-2 media and kept in the incubator. After a two-day incubation period, the second layer, consisting of endothelial cells, was cultured over the first layer at the top and bottom of the valve’s scaffold. To achieve this, the valve scaffold with the first layer underwent washing with Dulbecco’s phosphate buffered saline (DPBS 1X) and was placed back between the sterile PEEK mandrels. Endothelial cells (0.5 million cells) mixed with collagen type I (RatCol rat tail collagen type I 4 mg/mL, Advanced BioMatrix, Carlsbad, CA), were then injected into the H-TEHV using the mandrel apparatus. Following this step, the H-TEHV was incubated for 1.5 h at 37 °C without cell culture media to complete the culture of the second layer (Figure 5).

The H-TEHV was subsequently separated from the mandrel apparatus and transferred into a sterile Petri dish with a medium mixture (50% endothelial cell growth medium, 25% smooth muscle growth medium, and 25% fibroblast growth medium). The developed H-TEHV received a daily supply of fresh medium mixture (50% endothelial growth medium, 25% smooth muscle growth medium, and 25% fibroblast growth medium) until the day of implantation.

**Figure 6:**
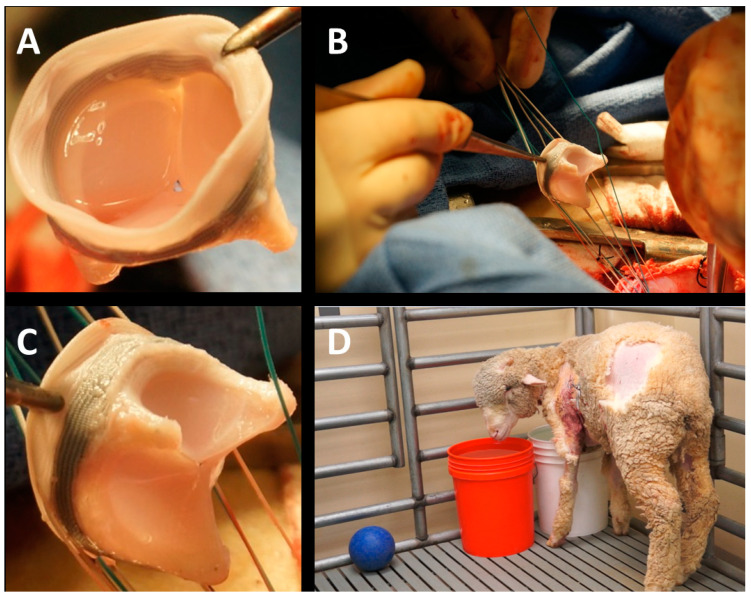
(**A**) An atrial view of the H-TEHV before surgical implantation into the sheep’s mitral position; (**B**) the H-TEHV during open-heart surgery, guided by suture lines into the mitral position; (**C**) a closer view of the H-TEHV during implantation, highlighting the suture lines; (**D**) the sheep post-recovery from anesthesia, standing on its feet without any signs of stroke.

**Surgical implantation of mitral H-TEHV in sheep:** Three weeks after the extraction of the jugular vein, the 21 mm H-TEHV was implanted in the same sheep’s mitral position through a midsternal thoracotomy using a cardiopulmonary bypass machine under the control of a perfusion team. The sheep weighed 45 kg on the day of surgery. The surgical procedure was performed according to the UCI IACUC protocol #2012-3071. The open-heart surgery was successfully led by Dr. Jeffrey C. Millikan and our surgical and perfusion teams at UCI Medical Center (Figure 6). We preserved the sheep’s native mitral valve’s anterior and posterior leaflets, and both were incorporated into the H-TEHV’s annular sutures. After completion of surgery, the animal was recovered from anesthesia and transferred on its feet to the UCI vivarium without any sign of stroke or other surgery-related problems (Figure 6D).

**Echocardiographic assessment:** During the procedure, the implanted H-TEHV was comprehensively monitored using echocardiography. A GE Vivid E9 echocardiography system (GE Healthcare, Milwaukee, WI) and a 4VD ultrasound transducer were used during the surgery. After the H-TEHV implantation, the mobility of the leaflets was studied based on B-mode imaging (Video S1). The color Doppler study was performed to test the presence of blood leaking or regurgitation through the H-TEHV valve (Video S2).

## 3. Results

**Durability of the H-TEHV’s scaffold:** After 50 million cycles in the AWT system, no defects were observed on the Carbothane valve leaflets, at the commissures between the leaflets, on the valve posts, or on the sutures and sewing ring.

**H-TEHV development:** The H-TEHV was successfully developed within a span of three weeks from a jugular vein extraction, with all tissue layers grown seamlessly on the valve scaffold. The top and bottom side views of the completed H-TEHV are presented in Figure 5B,C, revealing no defects in the tissue layers. Notably, there was no evidence of shrinkage or contraction in the tissue layers at the top and bottom sides of the valve scaffold. The tissue layers provided a uniform coverage over the valve scaffold, encompassing the leaflet’s belly and all edges, as shown in Figure 5B,C. Additionally, elongated cells were observed within the tissue layers using a light microscope, as illustrated in Figure 5D,E, over the Carbothane valve scaffold.

The thickness of the cultured valve’s leaflets, measured at approximately 0.6 mm prior to implantation, exhibited uniformity throughout the valve.

**Figure 7:**
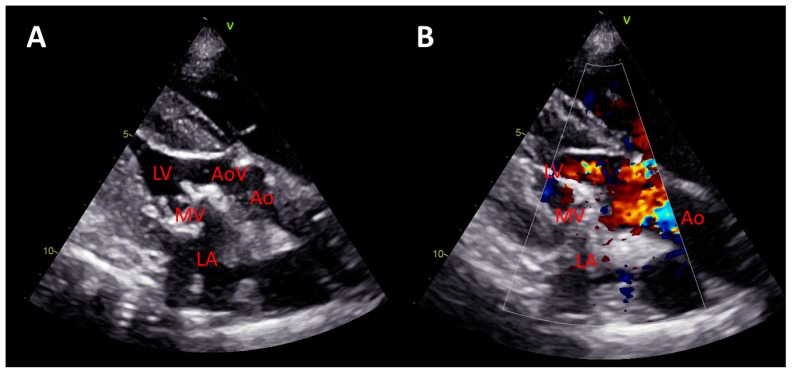
Transthoracic echocardiography of the sheep after hybrid valve implantation (**A**) The B-mode image reveals the proper implantation of the H-TEHV in the mitral position of the sheep, indicating favorable valve leaflet mobility with no observed restrictions. (**B**) A representation of the valve movement with a color Doppler flow pattern obtained from the H-TEHV implanted in the mitral position. The color Doppler image demonstrates the proper functioning of the valve, with no signs of leakage.

**Postimplantation imaging, and postmortem studies:** The B-mode echocardiography showed that the mobility of the valve’s leaflets was deemed favorable, exhibiting no signs of restriction (Figure 7A and Video S1). The color Doppler study further affirmed the competence of the H-TEHV, with no evidence of regurgitation observed (Figure 7B and Video S2). The planimetry measurements yielded a valve orifice area of 2.9 cm^2^, an ejection fraction (EF) of 67%, and a mean transmitral pressure gradient of 8.39 mmHg.

Upon the conclusion of the surgical procedure, the animal underwent a smooth recovery from anesthesia and was transferred to the UCI vivarium, displaying no indications of a stroke or other surgery-related complications. Unfortunately, the animal expired eight hours after being relocated to the vivarium. The cause of death was attributed to pulmonary edema induced by a mitral valve size mismatch, as indicated by the elevated mean transmitral pressure gradient.

**Figure 8:**
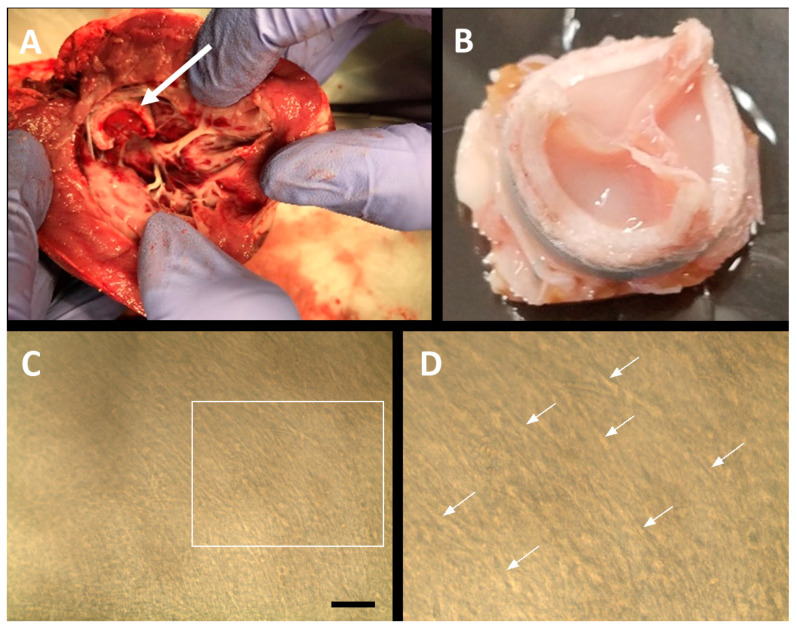
(**A**) Sheep heart dissection displaying the H-TEHV at the mitral position, indicated by a white arrow. (**B**) A top view of the H-TEHV post-dissection, demonstrating its integrity after 8 h of functioning in the sheep’s beating heart. (**C**) Macroscopic view revealing elongated FBs and SMCs in the initial tissue layer post-H-TEHV dissection. (**D**) A magnified view of the area within the white box in (**C**), highlighting elongated cells with white arrows. The scale bar for both images is 100 µm.

Postmortem examinations the day after the surgical procedure revealed the resilience of the H-TEHV, displaying no signs of tissue delamination or dehiscence, as illustrated in Figure 8A,B. Additionally, Figure 8C,D, captured from the surface of the H-TEHV after eight hours in the sheep’s mitral position, showcased the presence of elongated cells. These findings contribute valuable insights into the postimplantation performance and structural integrity of the developed H-TEHV.

## 4. Discussion

The overarching goal of eliminating the lifelong dependence on anticoagulation medication without compromising durability represents a significant stride toward enhancing the quality of life for younger patients in need of a heart valve replacement. Our focus has been on developing the H-TEHV with a permanent elastomeric scaffold, aiming to overcome the conventional drawbacks associated with both BHVs and MHVs. This approach seeks to mimic the biocompatibility and hemodynamics of a native valve while ensuring sufficient strength and durability. Elastomeric polymers, as heart valve leaflets, have demonstrated an outstanding hemocompatibility and durability, as evidenced in previous studies [22,23,24,25,26,27,28,29].

Given that heart valve replacement surgery is typically an elective procedure, most patients can afford to wait for approximately three weeks to have their autologous H-TEHV created, contingent upon the promising outcomes of the H-TEHV concept in the future.

This study marks our initial efforts, examining the feasibility of an autologous H-TEHV *in vivo* in an ovine model. The absence of regurgitation or leakage confirmed by echocardiography indicates the acceptable performance of the valve. Although no sign of mitral stenosis was observed in imaging, a mean transmitral pressure gradient of 8.39 mmHg indicated a mitral valve size mismatch. To avoid this, a larger valve should have been used for this animal.

The postmortem examinations further confirmed the integrity of the valve after eight hours of continuous function in the sheep, displaying no tissue separation or dehiscence, which addressed our primary concern related to the potential embolization of cultured tissue into the animal’s organs. Additionally, no traces of embolization were detected anywhere in the animal after a careful examination; the fact that the animal was able to walk without any signs of stroke potentially eliminates the likelihood of tissue embolization from the leaflets.

**Limitations:** This study represents an early but crucial step forward in assessing the feasibility of autologous H-TEHVs, paving the way for future advancements in tissue engineered heart valve technologies. A significant limitation of the present study is its reliance on a single animal study, a sample size that is insufficient for drawing definitive conclusions regarding the safety and effectiveness of H-TEHVs. Consequently, this study is best viewed as a case report, as it is crucial to share the ongoing progress and results, allowing others to replicate our efforts. The reporting of these findings serves the purpose of contributing to the collective knowledge in the field.

Another constraint in this study lies in the inability to characterize postimplant cells or tissues due to limitations posed by current standard microtomes, which are unsuitable for sectioning polymer-based leaflets. While our previous work successfully utilized a laser microtome for sectioning and analyzing nitinol-based hybrid tissue-engineered leaflets [16], this method could not be employed for the current scaffold. This limitation underscores the need for advancements in techniques for characterizing polymer-based structures, presenting an avenue for future research and innovation in the field.

## 5. Conclusions

We have successfully developed, tested, and, for the first time, implanted a novel H-TEHV featuring a permanent elastomeric scaffold in sheep. Our initial findings suggest that the H-TEHV demonstrates resilience under high-pressure conditions at the mitral position, exhibiting no signs of shrinkage or valve decomposition. While these short-term results may suggest the feasibility of the H-TEHV concept *in vivo*, it is crucial to acknowledge that given the current stage of development and the limited scope of a single animal implant, no definitive conclusions can be drawn regarding the long-term suitability of the H-TEHV.

Our forthcoming scientific objectives include the automation of the tissue culture technique through 3D printing [30]. Additionally, we aim to extend the duration of animal survival to twenty weeks, allowing for a thorough assessment of the performance and durability of H-TEHVs in chronic scenarios. To address a potential valve size mismatch, future experiments will incorporate cardiac CT scans upon the animal’s admission to our vivarium, ensuring accurate sizing for improved experimental precision.

## Data Availability

All the data that support the findings of this study are available upon reasonable request.

## Supplementary Materials

The following supporting information can be downloaded at: https://www.mdpi.com/article/10.3390/jcdd11020069/s1. Video S1: B-mode echocardiography shows the H-TEHV at the sheep’s mitral position with excellent leaflet motion. Video S2: The color Doppler study shows no sign of blood leakage or regurgitation through the mitral H-TEHV valve.
